# Auditory brainstem development of naked mole-rats (*Heterocephalus glaber*)

**DOI:** 10.1098/rspb.2022.0878

**Published:** 2022-08-10

**Authors:** Elizabeth A. McCullagh, John Peacock, Alexandra Lucas, Shani Poleg, Nathaniel T. Greene, Addison Gaut, Samantha Lagestee, Yalan Zhang, Leonard K. Kaczmarek, Thomas J. Park, Daniel J. Tollin, Achim Klug

**Affiliations:** ^1^ Department of Integrative Biology, Oklahoma State University, Stillwater, OK, USA; ^2^ Department of Physiology and Biophysics, University of Colorado Anschutz Medical Campus, Aurora, CO, USA; ^3^ Department of Otolaryngology, University of Colorado Anschutz Medical Campus, Aurora, CO, USA; ^4^ Department of Biological Sciences, University of Illinois at Chicago, Chicago, IL USA; ^5^ Department of Pharmacology, Yale University, New Haven, CT, USA; ^6^ Department of Cellular and Molecular Physiology, Yale University, New Haven, CT, USA

**Keywords:** naked mole-rat, auditory brainstem response, auditory, hearing onset

## Abstract

Life underground often leads to animals having specialized auditory systems to accommodate the constraints of acoustic transmission in tunnels. Despite living underground, naked mole-rats use a highly vocal communication system, implying that they rely on central auditory processing. However, little is known about these animals' central auditory system, and whether it follows a similar developmental time course as other rodents. Naked mole-rats show slowed development in the hippocampus suggesting they have altered brain development compared to other rodents. Here, we measured morphological characteristics and voltage-gated potassium channel K_v_3.3 expression and protein levels at different key developmental time points (postnatal days 9, 14, 21 and adulthood) to determine whether the auditory brainstem (lateral superior olive and medial nucleus of the trapezoid body) develops similarly to two common auditory rodent model species: gerbils and mice. Additionally, we measured the hearing onset of naked mole-rats using auditory brainstem response recordings at the same developmental timepoints. In contrast with other work in naked mole-rats showing that they are highly divergent in many aspects of their physiology, we show that naked mole-rats have a similar hearing onset, between postnatal day (P) 9 and P14, to many other rodents. On the other hand, we show some developmental differences, such as a unique morphology and K_v_3.3 protein levels in the brainstem.

## 1. Background

The naked mole-rat (*Heterocephalus glaber*) is the longest lived rodent, with lifespans reaching 30 years or longer [1–3]. One possible hypothesis for their longevity and other unique features is the retention of neonatal characteristics, such as adaptation for low oxygen tolerance [4–6] and protracted development in the hippocampus [7]. Whether this immaturity in adulthood is owing to altered development has not been well studied. Sensory systems, which have well-characterized developmental timing, provide the opportunity to assess developmental milestones.

One ideal model system to study developmental patterns is the sound localization circuit of the auditory brainstem, owing to its well-documented milestones [8]. This circuit is responsible for encoding sound localization information through timing and level differences between the two ears based on the frequency of the incoming auditory signal [9]. There is a significant body of literature on this neural pathway, and the biological significance, physiology and anatomy are all relatively well understood. For example, one key feature of auditory brainstem neurons is their ability to fire very rapidly (up to approximately 800 Hz) with high temporal precision. This is made possible by neuronal K_v_3 family voltage-dependent K^+^ channels, which promote high-frequency firing [10], and the K_v_3.3 channel is the most highly represented member of this family in auditory brainstem neurons [11]. Thus, any potential differences or similarities observed in naked mole-rats, such as anatomical markers for K_v_3.3, can be interpreted with relative ease. It is well established in several species and taxa that impaired or disordered peripheral development has effects on the neurons in the central nervous system which are downstream of the periphery [12,13] Therefore, as naked mole-rats may have prolonged or disordered peripheral auditory development [14], the question becomes whether central organization could also be impaired or changed in these animals. The study of the auditory brainstem's development might therefore give insight into altered development in the species.

Subterranean animals have very different hearing and sound localization needs, such as limited air-borne sound localization cues, as compared to animals living in above-ground environments. The hearing systems of many subterranean species are also altered in particular ways, including the absence of pinnae [15] or as in golden moles, the use of seismic communication [16]. Naked mole-rats lack pinnae, but they have developed a social lifestyle as well as a variety of social communication calls, which travel well in their burrows [17,18].

Naked mole-rats have high hearing thresholds and appear to be mostly sensitive to low-frequency sounds [17,19]. It was thought that this species had degenerate sound localization capabilities, particularly for high-frequency sounds, based on a diminished lateral superior olive (LSO) and poor performance in behavioural sound localization tasks [19]. The LSO plays a critical role in the neural encoding of interaural level difference cues to sound location of high frequencies. However, more recent studies demonstrated that mole-rats demonstrate an LSO with different, but perhaps not diminished morphology, as well as other auditory brainstem structures needed for proper sound localization ability [20]. Interestingly, while naked mole-rat anatomical auditory brainstem structures are present, they are lacking hyperpolarization-activated and nucleotide-gated channel 1 (HCN1) channels, perhaps contributing to their overall poor sound localization ability and increased auditory thresholds [20].

Hearing onset for most altricial rodent species is usually anywhere from postnatal day (P) 10–14 [21]. This is a critical time during auditory development where the brainstem and other areas undergo significant changes to become mature, and it has been shown that increased auditory stimulation or other manipulation during this time period can have lasting impacts on the auditory system [22,23]. It is unknown whether the naked mole-rat undergoes similar central organization during this time period, or if prolonged peripheral auditory development is also associated with prolonged central development [14].

This study aims to determine whether the naked mole-rat has similar auditory development to other commonly used rodent species (mouse and gerbil). These two other species were chosen because both are standard animal models for auditory research and thus a large body of background literature is available for comparison. Owing to their delayed peripheral auditory maturation [10] and long lifespan, we predicted changes in the maturation of both the anatomical and molecular organization of central auditory structures in naked mole-rats compared to other rodents. We aimed to investigate the (i) morphology of LSO and medial nucleus of the trapezoid body (MNTB) across development; (ii) K_v_3.3 expression during MNTB and LSO development; and (iii) hearing onset in naked mole-rats.

## 2. Methods

All experiments complied with all applicable laws, NIH guidelines, and were approved by the institutional animal care and use committees (IACUC) of University of Illinois at Chicago (no. 21-024), Oklahoma State University (no. 20-07), Yale University (no. 2022-07842) and University of Colorado Anschutz Medical Campus (no. 00617).

### (a) Animals

Naked mole-rats (*H. glaber*) of several ages, P9, P14, P21 and P335-390 (1 year), were obtained from a colony maintained at the University of Illinois Chicago. Mongolian gerbils (*Meriones unguiculatus)* and some mice (*Mus musculus)* at P9, P14, P21 and adulthood (P60–P90) used in anatomical experiments were maintained in a colony at the University of Colorado Anschutz Medical Campus. Additional mice of the same background (C57BL/6 J) were used for anatomy from a colony maintained at Oklahoma State University and Yale University. Anatomical measures (immunofluorescence and Western blotting) and auditory brainstem responses (ABRs) were performed in naked mole-rats while gerbils and mice were used only for anatomical measures (volume, K_v_3.3 expression, Nissl stain and Westerns).

### (b) Auditory brainstem response measurements

ABR measurements and analysis methods are described in previous publications [24–27]. Naked mole-rats were anaesthetized with a mixture of ketamine-xylazine (100 mg kg^−1^, 10 mg kg^−1^, respectively) administered via intraperitoneal injections. Once the animals did not respond to toe-pinch, indicating adequate anaesthesia, platinum subdermal needle electrodes were placed under the skin. The active electrode was placed between the ears (vertex) with the reference at the nape. A ground electrode was inserted in the foot of the animal.

Animals were presented with monaural click stimuli through custom-built ear bars. Stimuli were generated and evoked potentials recorded (sampled at 44.1 kHz and immediately downsampled by half) via an RME Fireface UCX sound card (RME Audio, Haimhausen, DE), controlled with custom-built MATLAB software. Sound was generated with TDT MF1 multi-field magnetic speakers (Tucker-Davis Technologies, FL, USA) with the sound being calibrated prior to presentation using Etymotic ER-7C probe microphones (Etymotic Research Inc, IL, USA). Signals from the electrodes were amplified (10 000×) and digitized using an ISO-80 preamplifer and headstage (World Precision Instruments Sarasota, FL USA).

Clicks were presented to both ears at a sound pressure level of 100 dB sound pressure level (SPL; peak, re: 1000 Hz tone) attenuated in steps of 10 dB for threshold measurements. Data were averaged over 1000–2600 repetitions per stimuli and filtered with a second-order Butterworth filter (cut-offs at 100 Hz and 2000 Hz). Peaks in the ABR waveform were examined offline within a latency range of 1–10 ms. The lowest SPL at which an ABR peak was detected was taken as the threshold SPL. For each animal tested, the threshold was determined from the ear with the lower threshold. Several animals, particularly in the youngest age groups, did not have a recognizable ABR waveform for any stimulus SPL and are included as ‘no signal (N.S.)’ datapoints.

### (c) Tissue preparation

Naked mole-rats, mice and gerbils were euthanized with an overdose of pentobarbital (120 mg kg^−1^) and transcardially perfused with phosphate-buffered saline (PBS; 137 mM NaCl, 2.7 mM KCl, 1.76 mM KH_2_PO_4_ and 10 mM Na_2_HPO_4_ Sigma-Aldrich, St Louis, MO) followed by 4% paraformaldehyde (PFA). Following perfusion, animals were decapitated, and brains dissected out and placed in 4% PFA overnight followed by continued submersion in PBS until slicing. Prior to slicing, brains were washed three times for 10 min each in PBS to remove any residual PFA. Brains were then submerged in 4% agarose (in PBS) for stability during cutting of the tissue. Brain tissue was sliced in 70 or 100 µm thick coronal sections containing the auditory brainstem nuclei using a Vibratome (Leica VT1000 s, Nussloch, Germany). For Western blotting, a separate group of animals (naked mole-rats, mice and gerbils) were used; animals were euthanized followed by immediate decapitation and removal of brain regions. For Western blotting, brains were placed immediately at −80°C before being shipped on dry ice for analysis.

### (d) Immunofluorescence

Ten–20 free-floating sections per animal were blocked in antibody media (AB media: 0.1 M phosphate buffer (PB: 50 mM KH_2_PO_4_, 150 mM Na_2_HPO_4_), 150 mM NaCL, 3 mM Triton-X, 1% bovine serum albumin) and 5% normal goat serum (NGS) for 1 h at room temperature on a laboratory shaker. Sections were stained with a primary antibody for K_v_3.3 (table 1) diluted in AB media and 1% NGS overnight at 4°C. Slices were then washed three times (10 min each wash) in PBS. Following washes, a secondary antibody (1 : 1000, goat anti-rabbit IgG (H + L) Thermo-Fisher, A-21429, RRID:AB_2535850 Alexa 555 or 1 : 1000 donkey anti-rabbit IgG (H + L) Thermo-Fisher, A32795, RRID: AB_2762835 Alexa 647) diluted in AB media and 1% NGS were applied for 1 h at room temperature. The secondary antibody was washed off similarly to primary antibodies (three washes, 10 min each wash) followed by the application of Nissl (Neurotrace 640/660 Deep-Red Fluorescent or Neurotrace 500/525 Green Fluorescent Nissl Stain, Invitrogen, Carlsbad, CA) diluted to a concentration of 1 : 100 in AB media for half an hour at room temperature. Lastly, sections were washed in PB, mounted on glass slides, fixed with Fluoromount-G (Southern Biotech, Cat. no.: 0100-01, Birmingham, AL) and coverslipped. Slides were stored in a 4°C fridge in a light-tight slide box until imaging.

**Table 1.** Primary antibodies are used in immunofluorescence/Western blotting.

antibody	immunogen	manufacturer, species, mono or polycolonal, cat. or lot no., RRID	concentration
anti-KCNC3 (K_v_3.3)	KSPITPGSRGRYSRDRAC (aas 701–718 of rat KCNC3)	Alomone (Jerusalem, Israel), rabbit polyclonal, APC-102, RRID: AB_2040170	1 : 1000 (immuno), 1 : 400 (Western)
GAPDH	genetic locus: GAPDH (human) mapping to 12p13.31	Santa Cruz (0411), mouse, monoclonal, cat. no. sc-32233, lot no. G3020	1 : 1000

### (e) Western blot

Brainstem tissues for mouse, gerbil and naked mole-rat were homogenized using a tissue grinder and 200 µl T-PER Tissue Protein Extraction Reagent (Thermo-Fisher, cat. no. 78510) that included a Roche complete Mini Protease inhibitor tablet (Roche, cat. no. 11836170001) and PhosSTOP Easypack (Roche, cat. no. 04906837001). Homogenized samples were shaken at 4°C for 30 min and centrifuged at 13 000*g* for 15 min (at 4°C) to remove large-cell debris. The supernatants were then aliquoted, quickly frozen in liquid nitrogen and stored at −80°C until use. Protein estimation was done using Bradford's reagent (Bio-Rad). Samples were suspended in 4× sample buffer for electrophoresis and incubated at room temperature for 30 min before loading on an SDS-PAGE gel. After electrophoresis, the protein was transferred onto polyvinylidenedifluoride membranes (Bio-Rad). Blots were then blocked in TBST (a mixture of tris-buffered saline and Tween-20) containing 5% non-fat milk for 1 h at room temperature with shaking. Blots were then incubated with the respective primary antibodies overnight at 4°C. After three washes with TBST, blots were incubated for 1 h with horseradish peroxidase-conjugated secondary antibodies, followed by extensive washes in TBST. Labelled proteins were detected by enhanced chemiluminescence. Polyclonal rabbit anti-Kv3.3 antibody (APC-102, Alomone Labs) was used at 1 : 400; monoclonal mouse anti-GAPDH antibody (sc-32233, Santa Cruz) was used at 1 : 1000.

### (f) Antibody characterization

A primary antibody was used for the detection of K_v_3.3 (listed in table 1) coupled with a fluorescent-conjugated secondary antibody. K_v_3.3 (APC-102, Alomone, Jerusalem, Israel; RRID: AB_2040170) is a rabbit polyclonal antibody specific to amino acids 701–718 of the rat KCNC3 (K_v_3.3) protein. K_v_3.3 channels are known to be highly expressed on the calyx of Held and LSO, which in adult animals, is consistent with what we see in the present study. Additionally, testing with K_v_3.3 knockout animals showed no immunoblot reactivity using this antibody [28].

### (g) Imaging

Brainstem sections were imaged using an Olympus FV1000 confocal microscope (Olympus, Tokyo, Japan) with lasers for 405, 543 and 635 nm imaging. High-resolution (1024 × 1024 pixel), 20× (UPLSAPO20X, NA 0.75) images were taken for sections displayed in the results at different developmental timepoints. Additional images were taken using a Zeiss (Jena, Germany) LSM 980 with Airyscan2 (1024 × 1024 pixel) at 20× (NA 0.8). The MNTB and LSO were characterized by their morphology, location and position in the trapezoid body and superior olive, and by the shape and size of neurons within these nuclei. Specifically, sections were cut from posterior–anterior and the seventh nerve and cochlear nuclei used as a reference for location of the MNTB and LSO. A montage, covering an extended field of view, was captured by imaging multiple 20× tiles and stitching them together using the Olympus FV10-ASW acquisition software.

### (h) Quantification of volume and plotting of data

Volumes of LSO and MNTB in naked mole-rats, gerbils and mice were quantified similar to previous work [29]. Briefly, two brainstems per species were stained for Nissl (as above) and imaged at 20× using the FV1000 or Airyscan2. Images were taken at a lower resolution (512 × 512 pixels) since only the extent of the LSO and MNTB were needed. The entire extent of the LSO and MNTB were imaged in the 70 or 100 µm thick sections and later outlined using Fiji [30]. The outline was then used to calculate the area of MNTB and LSO using the measure feature in Fiji. The volume of each nucleus was then estimated by multiplying each area measurement by 70 mms or 100 mms (the thickness of the slice) and summed to get the total volume for the nucleus. Both left and right MNTB and LSO were analysed for each animal resulting in four measurements per section (two MNTBs and two LSOs) of volume which were then used to calculate the values of mean and standard error reported here. Graphs were generated using R [31] ggplot2 [32]. Representative images were adjusted for brightness and contrast using Fiji purely for the purposes of illustration. All analyses were completed on unadjusted images only.

### (i) Statistical analysis

Statistical analyses were performed in R for the volume, ratio and Western blotting data comparing developmental timepoints within one species (one-way ANOVA) and between species (two-way ANOVA). Tukey's honest significant difference (HSD) tests were performed as *post hoc* analyses to make comparisons between species and timepoints. Tukey HSD includes a corrected *p*-value for multiple comparisons. Data are presented as mean +/− standard error. Where values are indicated as statistically significant: * = *p* < 0.05, ** = *p* < 0.01 and *** = *p* < 0.001.

Raw data for ABRs and volume measurements are available on Dryad [33].

## 3. Results

This study presents data from anatomical measures and auditory brainstem recordings at different developmental timepoints to establish whether the auditory brainstem of the naked mole-rat develops similarly to other commonly used rodent species in auditory neuroscience, specifically gerbils and mice.

### (a) Morphology of lateral superior olive and medial nucleus of the trapezoid body across development

**Figure 1.**
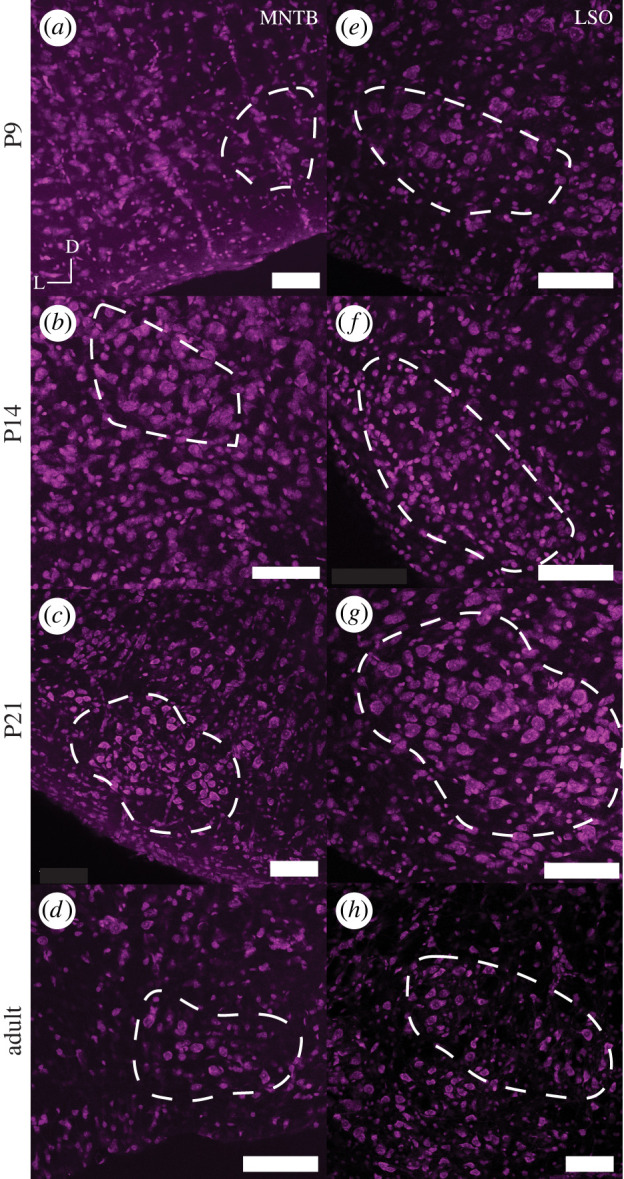
Nissl staining of MNTB and LSO in naked mole-rat at P9, P14, P21 and adult stages. Each panel represents a 20x image (scale bar is 100 microns) of the MNTB (*a–d*) and LSO (*e–h*) at different developmental timepoints. D represents the dorsal direction and L the lateral orientation of the brain slice. Outlined in white is the overall shape and boundaries of the MNTB or LSO. (Online version in colour.)

We compared the morphology and developmental maturation of the MNTB and LSO in naked mole-rats to mouse and gerbil. Similar to [20], when stained for Nissl, our results show that the LSO of naked mole-rats does appear to have a different shape (more oblong) compared to other rodents (which are typically more U- or S-shaped), and this shape is established early in development (P9) (figure 1*a*–*d*). Comparative data presented for gerbil and mouse show the distinct shape of the LSO across developmental timepoints (electronic supplementary material, figure S1a–l).

**Figure 2.**
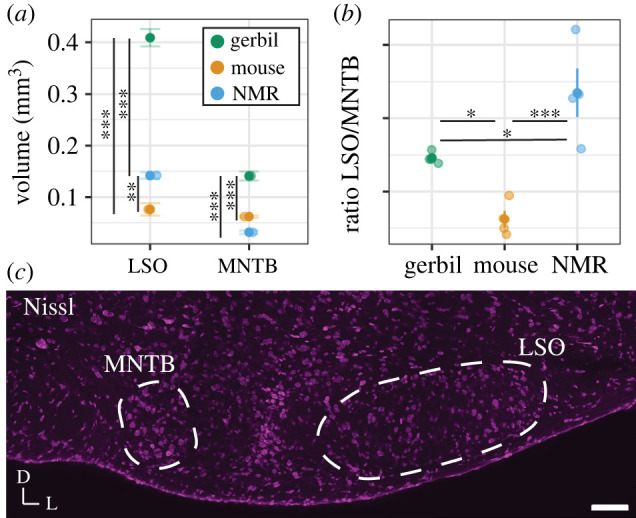
The comparison of volume of auditory brainstem structures between naked mole-rats, mice and gerbils. (*a*) Volume of the nuclei in the three species (gerbil, mouse and naked mole-rat (NMR)); (*b*) LSO/MNTB ratio for the three species, value of 1 indicated equal size between LSO and MNTB, greater than 1 is larger LSO to MNTB; and (*c*) Nissl montage image of the naked mole-rat auditory brainstem (scale bar 100 microns, MNTB and LSO outlined in white). (Online version in colour.)

MNTB can be seen in P9 with a consistent trapezoid-like shape at all developmental stages measured (figure 1*e*–*h*). Comparative data presented for gerbil and mouse show MNTB shapes across developmental timepoints (electronic supplementary material, figure S2a–l). These data suggest that MNTB is present and clearly distinguishable in all three species and at all ages imaged, despite the different lifestyles and different audiograms of the three species. To determine whether there are size differences in the volume of brainstem nuclei of naked mole-rats compared to mouse and gerbil, we measured the total volume of LSO and MNTB in the naked mole-rat. We found that despite physically being between a mouse and gerbil in terms of adult size, naked mole-rats showed a relatively small MNTB (closer in size to a mouse and significantly smaller than a gerbil (*p* < 0.001), figure 2*a*). By contrast, the volume of the LSO in naked mole-rats was in between the volume of mouse and gerbil and significantly different in volume from either (table 2, *p* < 0.01 mouse to naked mole-rat and *p* < 0.001 gerbil to naked mole-rat). To account for differences in size between these animals, and to measure the relative size of MNTB to LSO, we calculated the ratio of the LSO/MNTB volumes [34]. A value of 1 would indicate an equal size LSO to MNTB, whereas values greater than 1 indicate a larger LSO than MNTB. This ratio shows that the LSO of naked mole-rats is significantly larger than the MNTB, in comparison to gerbil (130% greater) and mouse (350% greater) (figure 2*b*).

**Table 2.** Descriptive statistics of volume measurements (mm^3^) for the three species. (*N* is number of animals, *n* = number of sections per nucleus, age of animals indicated next to each species.)

nucleus	naked mole-rat (P335–390)		mouse (P71)		gerbil (P60–72)	
	mean ± s.e.m.	*N* (*n*)	mean ± s.e.m.	*N* (*n*)	mean ± s.e.m.	*N* (*n*)
LSO	0.14 ± 0.006	2 (12–15)	0.076 ± 0.01	2 (7–11)	0.41 ± 0.02	2 (7–14)
MNTB	0.03 ± 0.003	2 (6–8)	0.06 ± 0.002	2 (11–13)	0.14 ± 0.009	2 (9–11)

### (b) K_v_3.3 expression during medial nucleus of the trapezoid body and lateral superior olive development

**Figure 3.**
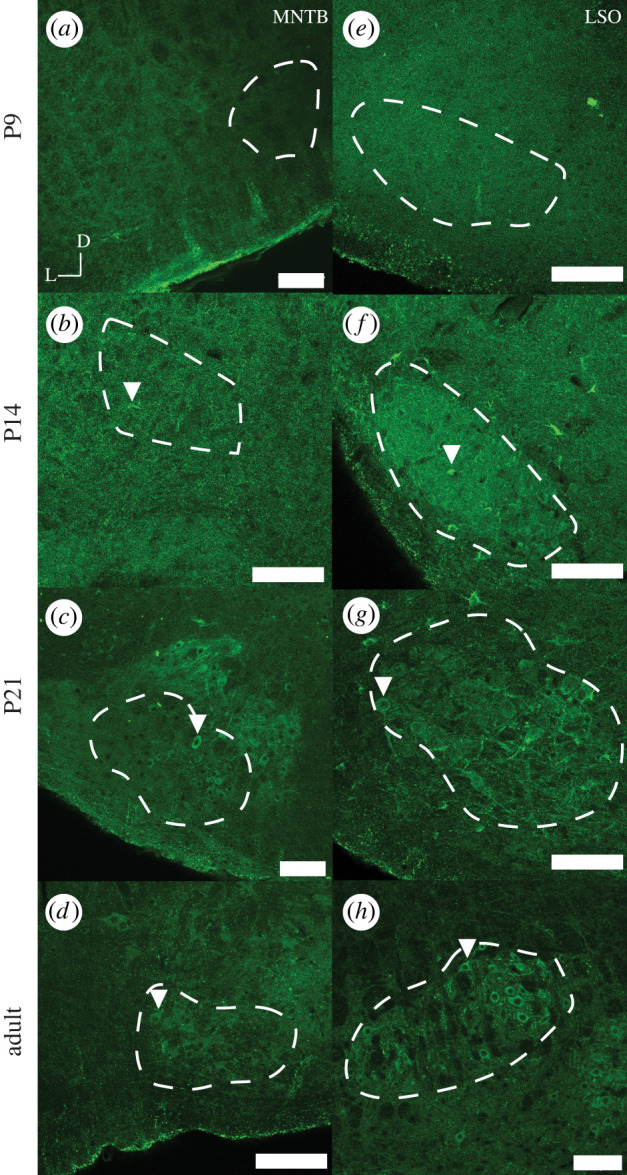
K_v_3.3 staining of MNTB and LSO in naked mole-rat at P9, P14, P21 and adult stages. Each panel represents a 20× image (scale bar is 100 microns) of the MNTB (*a–d*) and LSO (*e–h*) at different developmental timepoints. D represents the dorsal direction and L the lateral orientation of the brain slice. Outlined in white is the overall shape and boundaries of the MNTB or LSO. Arrowheads indicate an exemplar cell with adult-like K_v_3.3 expression pattern. (Online version in colour.)

**Figure 4.**
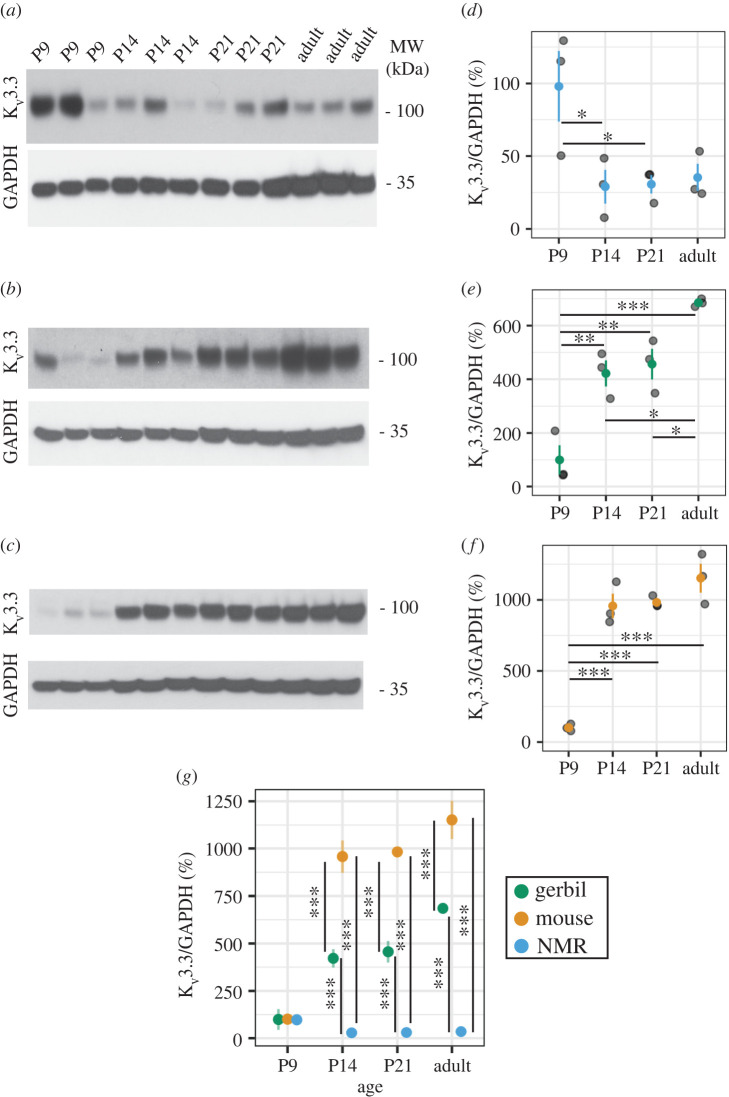
K_v_3.3 Western blotting of brainstem naked mole-rat (NMR), gerbil and mouse at P9, P14, P21 and adult stages. Blots containing samples for each animal are shown for brainstem (*a–c*) for both K_v_3.3 and GAPDH housekeeping proteins in naked mole-rat (*a,d*), gerbil (*b*,*e*) and mouse (*c,f*). Quantification of protein levels across animals is shown for each species and timepoint as a ratio of K_v_3.3/GAPDH. *N* = 3 for each time developmental timepoint for each species. Comparisons between species are shown in (*g*). (Online version in colour.)

We compared the expression pattern of the voltage-gated potassium channel K_v_3.3 through development in the MNTB and LSO of naked mole-rats with that in gerbils and mice (figure 3*a–d* MNTB, figure 3*e*–*h* LSO; figure 4; electronic supplementary material, figures S3 and S4). In gerbils and mice, there was relatively little or no immunostaining for K_v_3.3 at P9. Cellular expression increased at subsequent developmental stages (P14, P21 and adult) (electronic supplementary material, figures S3 and S4). Cellular expression consists of the expression of the antibody marker as puncta outside cells with some internalization of antibody in the cytoplasm, similar to that seen in other studies and denoted here with arrowheads [35,36]. The same pattern of increases during development was also observed on Western blots of total brainstem of gerbils and mice (figure 4*b,c*) and was quantified by densitometry (figure 4*e–g*).

The pattern of expression of K_v_3.3 in naked mole-rats appeared different from that in gerbils and mice. While the expression of K_v_3.3 was low at P9 and higher at the later developmental timepoints, there was no clear difference in cellular expression levels in either nucleus between P14, P21 and adult animals on immunostained sections of MNTB and LSO, whereas in gerbils and mice there is an increase in cellular expression of K_v_3.3 (figure 3). On Western blots of brainstem of naked mole-rats, there was also no significant difference between K_v_3.3 levels at P14, P21 and adult (figure 4*a,c* and table 3). When directly comparing protein levels between species, naked mole-rat K_v_3.3 protein was significantly lower than gerbil or mouse at all ages past P9 (*p* < 0.001, *N* = 3). In contrast with the immunostaining, however, higher levels of K_v_3.3 were detected at P9, compared to P14 and P21 on the immunoblots of naked mole-rats (*p* < 0.05, *N* = 3). These may potentially reflect the presence of K_v_3.3 channels in non-auditory regions of the brainstem of the naked mole-rat.

**Table 3.** Descriptive statistics of K_v_3.3 immunoblots. (Adult age for each species varied, naked mole-rats (P335–395), mouse (P90) and gerbil (P64).)

age (P)	naked mole-rat		mouse		gerbil	
	mean ± s.e.m.	*N*	mean ± s.e.m.	*N*	mean ± s.e.m.	*N*
9	98 ± 24.3	3	101.2 ± 14.0	3	99.7 ± 54.7	3
14	29 ± 11.6	3	957.7 ± 85.9	3	421.7 ± 48.5	3
21	30.7 ± 6.3	3	982.3 ± 23.4	3	456.3 ± 57.1	3
adult	35.3 ± 9.4	3	1152 ± 101.0	3	685 ± 8.1	3

### (c) Hearing onset in naked mole-rats

**Figure 5.**
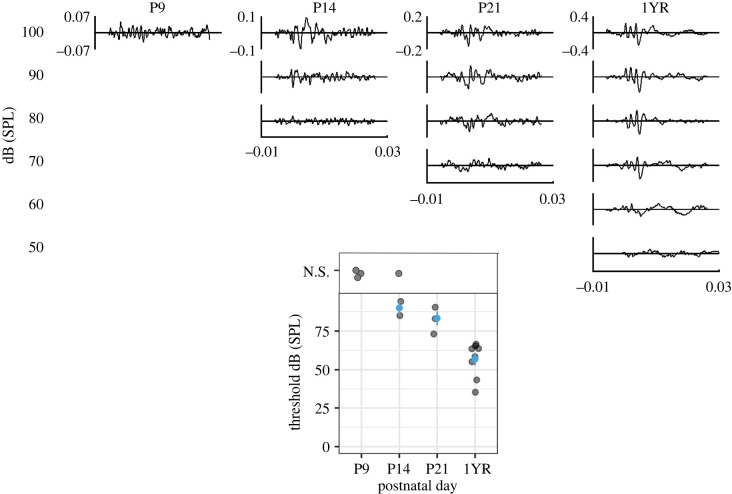
ABR measurements in naked mole-rats during development. Top panel shows representative ABRs at P9, 14, 21, and approximately 1-year-old (1YR or P335–395) animals at varying intensities. Inner *y*-scale is microvolts and *x*-scale in milliseconds. Bottom panel shows the summary data for ABR thresholds at the different age groups. If no ABR waveform was detected, the animal was given a datapoint of N.S. or ‘no signal’. (Online version in colour.)

Hearing onset is an important developmental milestone for rodents. We measured the ABR threshold at different developmental timepoints in naked mole-rats to determine ABR hearing onset. At P9, there was no ABR waveform distinguishable from the background of the ABR recording (figure 5 and table 4). However, at P14, in some animals (2 of 3), we were able to detect a signal, suggesting that hearing onset for these animals is probably between P9 and P14. As expected, naked mole-rats' ABR threshold decreased with age, with adult animals having the lowest thresholds at around 60 dB SPL.

**Table 4.** Descriptive statistics of ABR thresholds, responses averaged across animals (*N*).

age (P)	naked mole-rat		*N* (no signal)
	mean ± s.e.m.	*N* (signal)	
9	no signal	0	3
14	90 ± 5	2	1
21	83.3 ± 4.4	3	0
335–395	56.9 ± 2.7	8	0

## 4. Discussion

There are four main conclusions from this comparative study: (i) as shown previously, naked mole-rats have both a prominent MNTB and LSO, comparable to other rodents [20], LSO in naked mole-rats is largely elongated rather than U- or S-shaped; (ii) the ratio of LSO to MNTB is highest in naked mole-rats, indicating a relatively large LSO and a relatively small MNTB compared to the other rodent species; (iii) K_v_3.3 expression is low at P9 and there is no clear difference at other developmental timepoints whereas mice and gerbils have a progressive increase in expression patterns into maturity; and (iv) hearing onset in naked mole-rats is similar to hearing onset in other rodents. ABR auditory hearing onset for mouse [37] (P9–P12) and gerbil [38] (P10–P14) is comparable with our data showing a similar ABR hearing onset in naked mole-rats.

### (a) Morphology of lateral superior olive and medial nucleus of the trapezoid body across development

The LSO is important for processing of binaural sounds, particularly level differences between the two ears [9]. Historically, it was thought that owing to their underground environment and low-frequency hearing sensitivity, naked mole-rats had relatively fewer neurons in the LSO compared to other species [19]. Our study and more recent evidence indicate that the naked mole-rat LSO shows a slightly different oblong shape than other commonly studied rodent species [20]. The morphology and size of the LSO has been shown to depend in large part on the frequency range of hearing in mammals [39]. The prototypical ‘textbook’ LSO shape is the reverse ‘S’ shape, such as in the cat [40]. Cats have excellent low (less than approximately 100 Hz) and high (up to 60 kHz) frequency hearing, and each limb of the ‘S’-shaped LSO represents a different frequency range—low frequency on one limb of the ‘S’, mid frequencies in the middle limb and high frequencies in the opposite limb. The LSO is larger and more complex shaped in species with a large audiometric range, and smaller and simpler in species with a more restricted audiometric range [41]. For example, mice, rats and bats have even higher frequency hearing than cats but eliminated low-frequency hearing. As such, the LSO morphology is different from the prototypical cat ‘S’-shaped LSO by adding an additional high-frequency limb and losing the low-frequency portion of the limb of the ‘S’ shape. Mammals with more moderate ranges of hearing such as gerbils and guinea pigs have less sensitive high-frequency hearing and thus the LSO loses the high-frequency limb of the prototypical ‘S’ shape and becomes ‘U’ shaped. Finally, mammals with predominantly low-frequency hearing, such as human and elephant, retain only the low-frequency limb of the LSO thus exhibiting an oblong shape. Naked mole-rats have hearing restricted only to low frequencies [19]. Moreover, it has been shown previously that the cochlea of the naked mole-rat is fairly distinct, even in comparison to other mole-rats, in its low number of cochlear turns [42]. Therefore, the lack of a distinct shape in the LSO of naked mole-rats is consistent with this theory. Indeed, the LSO shape of naked mole-rats also closely resembles the ovoid shape found in many primates that also have predominantly low-frequency hearing similar to humans [21].

As expected, based on visual inspection, the MNTB of the naked mole-rat is relatively small, while the LSO, though lacking the familiar S-shape observed in many species, is relatively large. Based on their burrowing habitat, and previous work showing burrowers having large MNTBs [43], the prediction would be for naked mole-rats to have large MNTBs as well. However, as we and others have noted, naked mole-rats have a relatively small MNTB as compared to mouse and gerbil [20], though see [44], which might be associated with the limited hearing range of this species. To control for size variability between species in the volume measurements, and to specifically address auditory regions [34], we took a ratio of the LSO/MNTB volumes. This measurement clearly shows that in proportion to the size of the MNTB, the LSO of naked mole-rats is much larger in comparison to mouse and gerbil. Overall, our results for volume measurements across species are consistent with the published literature [45]. However, there may be strain differences in genetically inbred mice. For example, [29] reported an MNTB volume of 0.42 mm^3^ for CBA/CaJ mice while [46] and [45] reported LSO and MNTB of comparable volumes to our work (on C3HeB/FeJ and C57BL/6N backgrounds, respectively). While there is only one supplier of Mongolian gerbils (Charles River), there is probably substantial genetic divergence between various laboratory populations, including mouse strains, leading to some variation in LSO and MNTB volume measurements in these animals as well [47]. There are also fewer studies that cite LSO and MNTB volume measurements in gerbils for comparison of volumes reported, though our data are consistent with [48] and [49].

### (b) K_v_3.3 expression during medial nucleus of the trapezoid body and lateral superior olive development

The volume of the MNTB of naked mole-rats is small compared to gerbil or mouse. While the function of K_v_3.3 in this nucleus is likely to be conserved [35], we found differences in expression patterns during development from that in mice or gerbils. Overall, K_v_3.3 expression was relatively lower in naked mole-rats than in these other species. We saw clear K_v_3.3 expression in the naked mole-rat LSO that was consistent and relatively unchanging across development. Previous work has shown that the LSO weakly stains for K_v_3.3 gene expression, especially as compared to MNTB [44]. K_v_3.3, as well as other K_v_3 proteins, produce fast repolarization of cells following action potentials and this allows for accurate coding of high-frequency incoming stimuli [28]. In addition, K_v_3.3 channels are absolutely required for normal endocytosis of synaptic vesicles at presynaptic terminals on MNTB neurons, a result of the fact that they trigger the nucleation of the actin cytoskeleton at active zones [50]. Previous work has shown that the expression of K_v_3.1b (a related voltage-gated potassium channel to K_v_3.3) begins around P8–14 (mice), with mature expression observed at around P21 [48], but that another splice isoform of the channel, K_v_3.1a is expressed at much earlier developmental timepoints [51], if a similar alteration occurs with K_v_3.3, perhaps it occurs later in the naked mole-rat.

### (c) Hearing onset in naked mole-rats

Naked mole-rats have long lifespans [1] and have prolonged cochlear organization [14]; therefore, we expected that they would have a prolonged or delayed hearing onset as compared to gerbil and mouse [52]. However, our data suggest that naked mole-rats have similar hearing onset to gerbils (P10–P14) and mice (P9–P12) [37,38]. However, note that naked mole-rats do appear to have prolonged hearing development as evidenced by high ABR thresholds that persist to at least P21, limiting our results to suggesting that hearing onset occurs somewhere between P9 and P21. Thresholds reached their lowest values by P270+, suggesting mature adult-like hearing thresholds occur beyond P21, whereas in mice and gerbils, by P21 thresholds are already at adult values [37,38]. When during development, adult-like thresholds arise in naked mole-rats is an open question.

## 5. Conclusion

We have shown that hearing onset of the naked mole-rat is similar to that of mice and gerbils, while showing distinct morphology in brainstem regions. Specifically, the morphology of brain regions, as well as hearing onset as measured by ABR, occur at similar timepoints in all three species. By contrast, ABR measures indicate prolonged immature hearing thresholds in naked mole-rats and K_v_3.3 protein abundance and cellular localization are different compared to mice and gerbils. Further studies that show naked mole-rat LSO and MNTB neuronal response properties would help to determine the physiological relevance of both the ABR responses seen here (in terms of high thresholds) and functional significance of K_v_3.3 channel expression.

## Data Availability

Data are accessible through Dryad: https://doi.org/10.5061/dryad.tqjq2bw22 [33]. Electronic supplementary material, figures: https://figshare.com/s/df4805f194a36859f04c [53].
